# Large tumor suppressor 2 is a prognostic biomarker and correlated with immune infiltrates in colorectal cancer

**DOI:** 10.1080/21655979.2021.1996513

**Published:** 2021-12-19

**Authors:** Chengwen Zhao, Jianping Chen, Yonghui Liu, Shaoqing Ju, Guihua Wang, Xudong Wang

**Affiliations:** a Department of Laboratory Medicine, Affiliated Hospital of Nantong University, Nantong, Jiangsu, China; b School of Public Health, Nantong University, Nantong, Jiangsu, China

**Keywords:** Colorectal cancer, LATS2, prognosis, immune infiltration, biomarker

## Abstract

Colorectal cancer (CRC) is a common malignancy that has both low 5-year survival and high prevalence. Immunotherapy has achieved impressive progress for treatment of CRC, but still faces huge challenges. Although large tumor suppressor 2 (LATS2) is well accepted to be related to cancer progression, the prognostic potential and immune response role of LATS2 expression in CRC remain unclear. To investigate the value of LATS2 for prognosis and immune infiltration, a retrospective study of 213 CRC patients was carried out. We determined the expression of LATS2 in tumor tissues by immunohistochemistry. The results indicated that LATS2 expression was down-regulated in CRC tissues and clearly related to tumor differentiation (*P* = 0.002) and TNM stage (*P* = 0.002). Low LATS2 expression and TNM stage were subsequently identified as significant independent predictors of prognosis in CRC by univariate and multivariate analyses. In Kaplan–Meier survival analyses, CRC patients with elevated LATS2 expression and early TNM stage had better overall survival. We further found that LATS2 was involved in the regulation of immune-related pathways and that its expression was positively related to tumor-infiltrating immune cells by GSEA, TIMER, and ssGSEA analyses. In summary, our data imply that LATS2 may act as a cancer suppressor gene and be correlated with clinical prognosis and immune infiltration in CRC. Thus, LATS2 may be applied as a novel biomarker for predicting clinical outcomes and immune infiltration levels in CRC.

## Introduction

Colorectal cancer (CRC), including colon cancer (COAD) and rectal cancer (READ), is the leading cause of cancer-associated death worldwide and ranks third in morbidity and mortality for both men and women [1]. Despite remarkable advances in treatment methods, including surgery, chemotherapy, and radiotherapy, in recent years, the trends for 5-year survival of CRC patients remain poor [2,3]. Currently, the immune microenvironment plays a decisive part in the treatment and prognosis of CRC patients [4]. Many studies have shown that multiple immune microenvironmental factors have definite influences on the prognosis of CRC patients and thus immunotherapy is a hopeful direction for CRC treatment [5,6]. However, clinical trials revealed that some CRC patients were not sensitive to existing immunotherapies, indicating that they had a strong mechanism of immunological tolerance [7]. Therefore, the discovery and identification of new prognostic markers and immunotherapeutic targets in CRC is of paramount importance.

Large tumor suppressor 2 (LATS2), a member of the LATS tumor suppressor family, has important effects on the regulation of cell growth, proliferation, and apoptosis [8]. Dysregulated expression of LATS2 may be involved in the origin and progression of various malignant cancers [9,10]. For example, LATS2 expression was reduced and negatively related to tumor size and metastasis in breast cancer [11]. In hepatocellular carcinoma, LATS2 mediated yes-associated protein (YAP) phosphorylation and promoted nuclear localization of YAP1 and YAP1/TEAD2 interactions, which in turn induced cancer progression [12]. Furthermore, LATS2 was a tumor suppression gene in ovarian cancer and decreased LATS2 led to upregulation of PD-L1, which subsequently promoted T cell apoptosis and suppressed NK cell function [13]. These results indicate that LATS2 has important functional roles in the development of cancer. However, the significance for LATS2 in cancer progression and immunology in CRC remains elusive.

In the present study, we hypothesized that LATS2 plays an important role in CRC progression and immunology. The aims of the study were not only to clarify LATS2 mRNA and protein expression in CRC patients by quantitative real-time PCR (qRT-PCR) and tissue microarray (TMA) immunohistochemistry analyses, respectively, but also to determine the correlations of LATS2 with clinicopathological characteristics and evaluate its prognostic role in these patients. Moreover, we identified the involvement of LATS2 in immune-related pathways and investigated its correlations with tumor-infiltrating immune cells.

## Materials and methods

### Patients and tissue specimens

A total of 533 specimens were collected for the study. Fresh surgical samples, comprising 27 CRC tissues and 27 matched normal tissues, were provided. Another 479 formalin-fixed, paraffin-embedded tissue samples, comprising 213 cancers, 174 matched normal surgical margins, and 92 benign tissues, were collected from CRC patients who underwent surgical therapy. All samples were provided by the Affiliated Hospital of Nantong University between 2009 and 2019 and were confirmed by two pathologists. Relevant clinical and pathological characteristics were recorded, including sex, age, tumor location, differentiation, TNM stage, primary tumor (T), lymph node metastases (N), distant metastases (M), and preoperative carcinoembryonic antigen (CEA) level. The patients ranged in age from 20 to 81 years. Before surgery, no patients had received any specific treatments such as immunotherapy, radiotherapy, or chemotherapy. Overall survival (OS) was measured from initial diagnosis to date of death. The study protocol was approved by the Human Research Ethics Committee of the Affiliated Hospital of Nantong University, Jiangsu, China. All participating patients provided written informed consent to use their tissues and publish the present study.

### TMA construction and immunohistochemistry analysis

TMA assembly was performed with a manual Tissue Microarrayer System (Quick-Ray, UT06; UNITMA, Seoul, South Korea). A standard protocol for immunohistochemistry was performed to examine the LATS2 protein expression levels in the 479 tissue blocks [14]. Briefly, the slides were incubated with an anti-LATS2 rabbit antibody (1:200 dilution; ab111054; Abcam, Cambridge, MA, USA) at 4°C overnight, further incubated with a rabbit anti-goat secondary antibody (1:200 dilution; Abcam) for 30 min at room temperature, and then stained with 3,3′-diaminobenzidine plus (Dako, Carpinteria, CA, USA). The LATS2 protein expression levels were scored using a Vectra 3.0 Automated Quantitative Pathology Imaging System (PerkinElmer, Waltham, MA, USA), with the staining intensity scored as 0 – (no staining), 1+ (weakly positive), 2+ (moderately positive), or 3+ (strongly positive). The final staining score was calculated by multiplying the percentage of stained cells by the intensity score, and ranged from 0 to 300. A cutoff point for the LATS2 expression score was estimated using the X-tile software [15].

### Cell lines and cell culture

A human normal intestinal epithelial cell line (NCM460) and four human CRC cell lines (DLD1, SW480, SW620, and HT29) were acquired from the Chinese Academy of Sciences (Shanghai, China). All cell lines were maintained in DMEM (Gibco, Grand Island, NY, USA) containing 10% fetal bovine serum (Gibco) at 37°C in a 5% CO_2_ incubator.

### qRT-PCR analysis

TRIzol reagent (Invitrogen, Carlsbad, CA, USA) was utilized to extract total RNA from CRC cell lines and tissues, and SuperScript® III Reverse Transcriptase (Invitrogen) was used to synthesize cDNA based on the manufacturer’s protocol. Next, qRT-PCR was carried out in an ABI 7500 quantitative PCR instrument (TaKaRa, Dalian, China). The primer sequences for LATS2 and GAPDH are listed in Table S1. GAPDH was amplified for normalization. Relative LATS2 expression was calculated by the 2^−ΔΔCT^ method.

### Gene set enrichment analysis (GSEA)

GSEA (http://gepia.cancer-pku.cn/) [16,17] was used to identify the enriched pathways that were related to LATS2 in CRC. The ‘c2.cp.kegg.v7.1.sym-bols.gmt’ was obtained from the MSigDB database [18]. Both nominal *p*-value <0.05 and *q*-value <0.25 were considered statistically significant.

### Tumor immune estimation resource (TIMER)

The TIMER (https://cistrome.shinyapps.io/timer) [19,20] is a database for systematic analysis of tumor-infiltrating immune cells across various cancers. The abundances of immune infiltration were estimated for CD4 + T cells, B cells, CD8 + T cells, macrophages, dendritic cells, and neutrophils.

### The GeneMANIA dataset

The GeneMANIA dataset (http://genemania.org) [21] is an integrated network of human protein-protein interactions that can annotate genomic and genetic datasets through data integration and thorough quality control. In the present study, we used this dataset to perform an analysis on the protein-protein interaction (PPI) network of LATS2.

### Statistical analysis

The associations between LATS2 protein expression and clinicopathological features were evaluated using the Pearson χ^2^ test. Survival curves were plotted by the log-rank test and subjected to Kaplan–Meier survival analysis. Univariate and multivariate Cox regression analyses were utilized to analyze prognostic factors of LATS2 in CRC. In addition, we used R language 3.6.3 version (https://cran.r-project.org) to identify LATS2 expression, its activated pathways, and its associations with immune infiltrate levels. All data involving immunohistochemistry were analyzed using the SPSS 20.0 statistical software package (SPSS Inc., Chicago, IL, USA). A *P*-value <0.05 was considered statistically significant.

## Results

In this study, we found that LATS2 mRNA expression was decreased in CRC tissues and cell lines. We then determined LATS2 protein expression and the relationships between LATS2 expression and clinicopathological parameters in CRC patients by immunohistochemical analysis, and performed univariate and multivariate analyses that identified LATS2 as an independent predictor of OS. Our bioinformatics analyses further indicated that LATS2 may be involved in multiple immune-related pathways and that its expression was positively correlated with most types of immune cells.

### LATS2 mRNA expression is decreased in CRC tissues and cell lines

**Figure 1.**
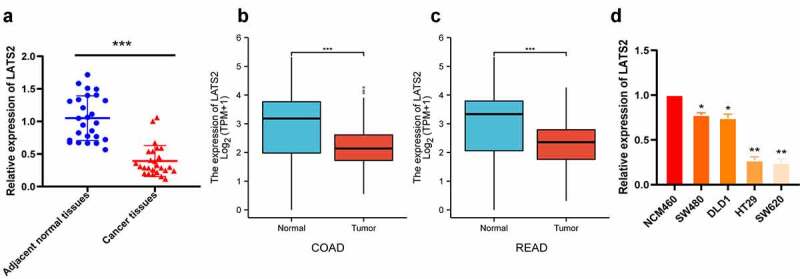
LATS2 mRNA expression in CRC tissues and cell lines. (a) LATS2 mRNA expression was significantly decreased in CRC tissues compared with matched adjacent normal tissues. (b-c) In the TCGA database, LATS2 mRNA expression was markedly lower in COAD and READ tissues compared with normal tissues. (d) LATS2 expression was decreased in CRC cell lines compared with a normal intestinal epithelial cell (NCM460). Data were expressed as mean ± SD, *P < 0.05; **P < 0.01; ***P < 0.001

The expression levels of LATS2 in 27 fresh CRC tissues and 27 matched adjacent normal tissues were evaluated by qRT-PCR. LATS2 expression was obviously decreased in CRC tissues compared with paired noncancerous tissues (*P* < 0.001; Figure 1(a), in accordance with The Cancer Genome Atlas (TCGA) database (Figure 1(b,c)). Furthermore, LATS2 expression was significantly decreased in CRC cell lines (*P* < 0.05; Figure 1(d)).

### Low LATS2 protein expression levels in CRC tissues

**Figure 2.**
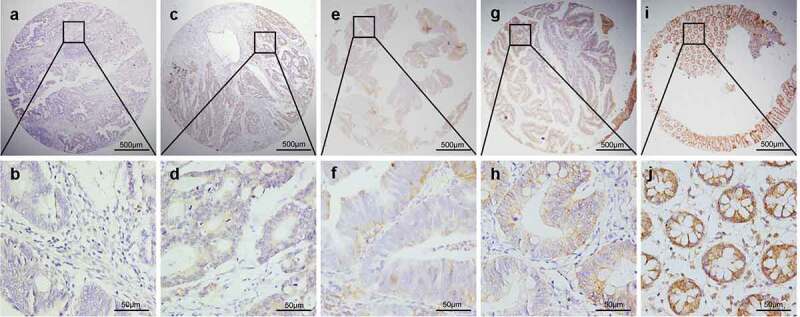
Representative patterns of LATS2 protein expression in colorectal benign and malignant tissues on TMA sections. (a–b) Colorectal cancer with low or no LATS2 expression. (c–d) Colorectal cancer with high LATS2 expression. (e–f) High-grade intraepithelial neoplasia with low LATS2 expression. (g–h) Low-grade intraepithelial neoplasia with low LATS2 expression. (i–j) Normal surgical margin of colorectal cancer with high LATS2 expression

The LATS2 protein expression levels in colorectal tissues were investigated by immunohistochemical analysis. Positive LATS2 staining was mostly observed in the cell membrane and cytoplasm with no nuclear staining in the examined tissues (Figure 2). We identified a significant cutoff point (180) for determining low or high LATS2 expression using the X-tile software. Thus, scores from 0 to 180 were considered low LATS2 expression, while scores from 181 to 300 were considered high LATS2 expression. Among the 479 archived tissue blocks, 56.32% of adjacent non-tumor tissues had high LATS2 expression, compared with only 23.08% of chronic colitis tissues, 15.91% of low-grade intraepithelial neoplasia tissues, 9.09% high-grade intraepithelial neoplasia tissues, and 33.80% of cancerous tissues (Table 1). Low LATS2 protein expression was mostly frequently observed in CRC tissues (Pearson χ^2^ = 45.401, *P* < 0.001).

**Table 1.** LATS2 expression in colorectal benign and malignant tissues

Characteristic	n	LATS2 High expression (%)	Low or no expression (%)	Pearson χ^2^	*P* value
Chronic colitis	26	6 (23.08)	20 (76.92)		
Low-grade intraepithelial neoplasia	44	7 (15.91)	37 (84.09)		
High-grade intraepithelial neoplasia	22	2 (9.09)	20 (90.91)		
Cancer	213	72 (33.80)	141 (66.20)		
Surgical margin ^a^	174	98 (56.32)	76 (43.68)		
Total	479	185 (38.62)	294 (61.38)	45.401	<0.001***

^a^Epithelium without intraepithelial neoplasia from colorectal cancer. ***P < 0.001.

### Associations of LATS2 expression with clinicopathologic parameters in CRC

The clinicopathological parameters of the 213 CRC patients are shown in Table 2. Low LATS2 expression was significantly related to tumor differentiation (*P* = 0.002), TNM stage (*P* = 0.002), primary tumor (T; *P* = 0.041), lymph node metastases (N; *P* = 0.012), and distant metastases (M; *P* = 0.006). The frequency of LATS2 expression was higher in early-stage CRC compared with advanced-stage CRC. No significant associations were found between LATS2 expression and sex, age, location, and preoperative CEA level (*P* > 0.05).

**Table 2.** Associations of LATS2 protein expression with clinicopathological characteristics in colorectal cancer patients

Characteristic	n	LATS2 High expression (%) Low or no expression (%)		Pearson χ^2^	*P* value
Total	213	72 (33.80)	141 (66.20)		
Sex				0.627	0.428
Male	135	43 (31.85)	92 (68.15)		
Female	78	29 (37.18)	49 (62.82)		
Age				0.041	0.839
<60	72	25 (34.72)	47 (65.28)		
≥60	141	47 (33.33)	94 (66.67)		
Location				2.712	0.100
Colon	160	59 (36.88)	101 (63.12)		
Rectum	53	13(24.53)	40 (75.47)		
Differentiation				12.555	0.002**
Poor	22	0 (0)	22 (100)		
Well and middle	177	67 (37.85)	110 (62.15)		
Others ^a^	14	5 (35.71)	9 (64.29)		
TNM stage				12.216	0.002**
0-I	46	23 (50.00)	23 (50.00)		
II	85	32 (37.65)	53 (62.35)		
III+IV	82	17 (20.73)	65 (79.27)		
T				4.156	0.041*
Tis+T1+ T2	53	24 (45.28)	29 (54.72)		
T3,4b	160	48 (30.00)	112 (70.00)		
N				10.972	0.012*
N0	134	53 (39.55)	81 (60.45)		
N1a	39	9 (23.08)	30 (76.92)		
N1b	21	7 (33.33)	14 (66.67)		
N2a,b	19	1 (5.26)	18 (94.74)		
M				7.652	0.006**
M0	199	72 (36.18)	127 (63.82)		
M1a+1b	14	0(0)	14 (100)		
Preoperative CEA (ng/ml)				1.263	0.532
≤5	131	48 (36.64)	83 (63.36)		
>5	26	8 (30.77)	18 (69.23)		
unknown	56	16 (28.57)	40(71.43)		

^a^Mucinous adenocarcinoma, 14 cases. *P < 0.05; **P < 0.01

### Low LATS2 protein expression predicts poor prognosis in CRC patients

**Figure 3.**
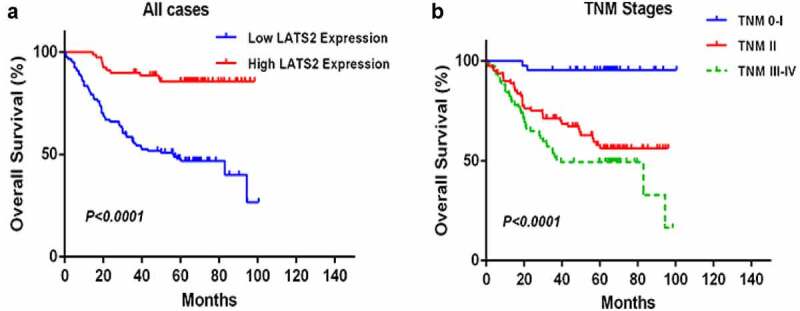
Survival analysis of CRC patients by the Kaplan–Meier method. (a) Overall survival in patients with high LATS2 expression was significantly higher than that in patients with low LATS2 expression. (b) Overall survival in patients with stage II or stage III–IV CRC was significantly lower than that in patients with stage 0–I CRC

Univariate and multivariate analyses were performed to determine the clinical prognostic value of LATS2 in CRC (Table 3). In the univariate analyses, LATS2 expression (HR: 0.087, 95% CI: 0.035–0.216, *P* < 0.001), tumor differentiation (HR: 2.672, 95% CI: 1.630–4.379, *P* < 0.001), TNM stage (HR: 2.193, 95% CI: 1.575–3.054, *P* < 0.001), T stage (HR: 6.693, 95% CI: 2.705–16.560, *P* < 0.001), N stage (HR: 1.479, 95% CI: 1.213–1.804, *P* < 0.001), M stage (HR: 6.418, 95% CI: 3.470–11.869, *P* < 0.001), and preoperative CEA level (HR: 2.593, 95% CI: 1.501–4.478, *P* = 0.001) were significantly correlated with OS. In the multivariate analyses, low LATS2 expression (HR: 0.151, 95% CI: 0.053–0.429, *P* < 0.001) and TNM stage (HR: 1.922, 95% CI: 1.377–2.993, *P* = 0.048) were identified as independent predictors of OS. Subsequent Kaplan–Meier survival analyses revealed that the survival time of CRC patients with high LATS2 expression was significantly prolonged (*P* < 0.0001; Figure 3(a)). In addition, CRC patients with early TNM stage had markedly longer OS than patients with advanced TNM stage (*P* < 0.0001; Figure 3(b)).

**Table 3.** Univariate and multivariate analyses of prognostic factors for overall survival in colorectal cancer patients

	Univariate analysis			Multivariate analysis		
	HR	*p*>|z|	95%CI	HR	*p*>|z|	95%CI
LATS2 expression High vs. Low	0.087	<0.001***	0.035–0.216	0.151	<0.001***	0.053–0.429
Age (years) ≤60 vs. >60	0.954	0.840	0.602–1.510			
Sex Male vs. Female	1.293	0.283	0.809–2.067			
Location Colon vs. Rectum	1.388	0.174	0.866–2.227			
Differentiation Poor vs. Well and middle	2.672	<0.001***	1.630–4.379	1.411	0.251	0.783–2.543
TNM stage 0-I vs. II vs. III+IV	2.193	<0.001***	1.575–3.054	1.922	0.048*	1.377–2.993
T Tis+T1+ T2 vs. T3, 4b	6.693	<0.001***	2.705–16.560			
N N0 vs. N1a vs. N1b vs. N2a, b	1.479	<0.001***	1.213–1.804			
M M0 vs. M1a+1b	6.418	<0.001***	3.470–11.869			
Preoperative CEA, ng/ml ≤5 vs. >5	2.593	0.001**	1.501–4.478	2.089	0.052	1.066–4.093

Abbreviation: HR, hazard ratio; CI, confidence interval. *P < 0.05; **P < 0.01; ***P < 0.001.

### LATS2 is involved in multiple immune-related pathways

**Figure 4.**
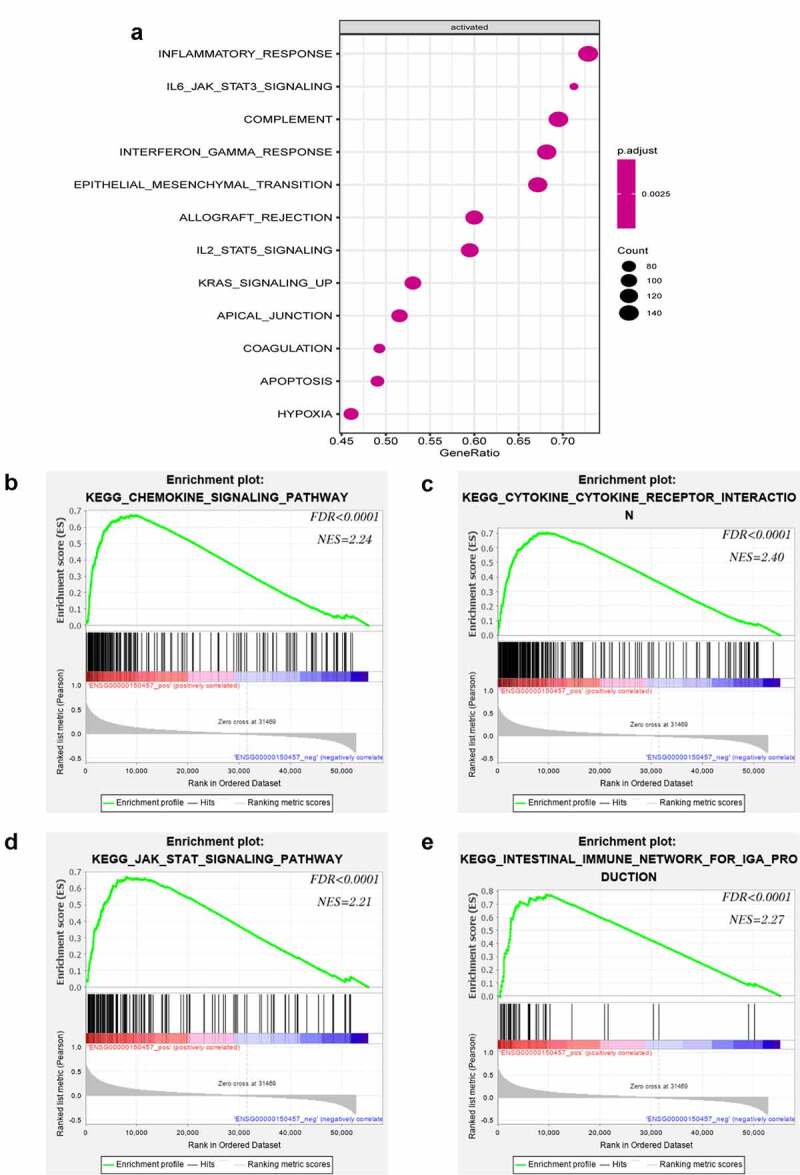
Notable immune-related signaling pathways in the high LATS2 expression group. (a) LATS2-activated signaling pathways. (b) Chemokine signaling pathways. (c) Cytokine-cytokine receptor interaction signaling pathways. (d) JAK-STAT signaling pathways. (e) Intestinal immune network for IgA production signaling pathways

Abbreviation: FDR, false discovery rate; NES, normalized enrichment score.

To elucidate the key role of LATS2 in CRC, we used R language to identify LATS2-related activated pathways. Surprisingly, inflammation and immune-related pathways including inflammatory response pathways, IL6-JAK-STAT3 signaling pathways, complement signaling pathways, and interferon-gamma response signaling pathways were significantly activated (Figure 4(a)). GSEA was further performed to identify pathways associated with LATS2 in CRC. Intriguingly, the results suggested that high LATS2 expression was strongly related to immune-related pathways, such as chemokine signaling pathways, cytokine-cytokine receptor interaction signaling pathways, JAK-STAT signaling pathways, and intestinal immune network for IgA production signaling pathways (Figure 4(b-e)).

### Correlations between LATS2 expression and immune infiltration

**Figure 5.**
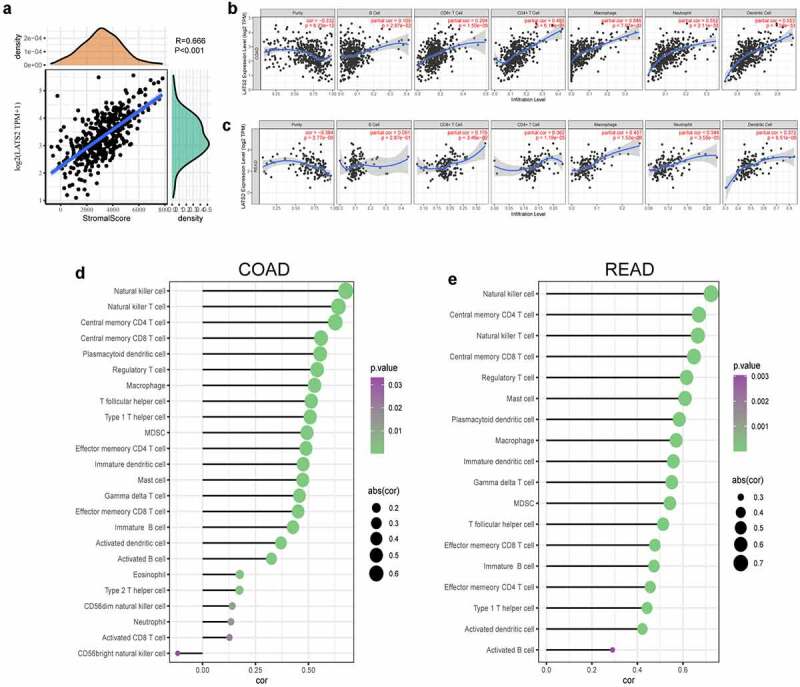
Correlations of LATS2 expression with tumor microenvironment and immune infiltration levels in CRC. (a) Positive correlation between LATS2 expression and immune score in CRC. (b) LATS2 was positively correlated with tumor-infiltrating immune cells in COAD by TIMER. (c) LATS2 was positively correlated with tumor-infiltrating immune cells in READ by TIMER. (d) LATS2 was positively correlated with tumor-infiltrating immune cells in COAD by ssGSEA. (e) LATS2 was positively correlated with tumor-infiltrating immune cells in READ by ssGSEA

Immune infiltration levels in the tumor microenvironment have crucial effects on survival and prognosis in many cancer patients. To explore the role of the LATS2-influenced immune microenvironment, we analyzed the immune scores in CRC samples and found that LATS2 expression had a significant positive association with immune score (*r* = 0.666, *P* < 0.001; Figure 5(a)). Next, the underlying relationships between LATS2 expression and tumor-infiltrating immune cells in CRC were investigated using the TIMER database. We discovered that LATS2 expression was negatively associated with tumor purity in COAD (*r* = −0.332, *P* = 6.23e−12) and READ (*r* = −0.384, *P* = 2.77e−6). However, LATS2 expression had positive correlations with several types of infiltrating immune cells, including B cells (*r* = 0.109, *P* = 2.87e−2), CD8 + T cells (*r* = 0.294, *P* = 1.50e−9), CD4 + T cells (*r* = 0.483, *P* = 6.10e−25), macrophages (*r* = 0.546, *P* = 7.97e−33), neutrophils (*r* = 0.552, *P* = 2.11e−33), and dendritic cells (*r* = 0.553, *P* = 1.39e−33) in COAD (Figure 5(b)). Similar findings were obtained in READ, except that LATS2 expression in READ had no significant association with B cell infiltration (Figure 5(c)). To validate these findings, we used the ssGSEA method and found strong positive correlations between LATS2 expression and natural killer cells, T cells, macrophages, and dendritic cells in CRC (Figure 5(d,e)). The relationships between LATS2 expression and vital gene markers for immune cells were also evaluated using the TIMER database (Table 4). The analysis revealed that LATS2 expression was positively related to the majority of gene markers for infiltrating immune cells in CRC. Strikingly, gene markers for CD8 + T cells, T follicular helper (Tfh) cells, and dendritic cells, such as PTPRC, BCL6, NRP1, and THBD, had stronger positive correlations with LATS2 expression than other gene markers in CRC. We further analyzed the correlations of LATS2 expression with these four gene markers using TCGA datasets, and found similar results (Figure S1). Taken together, LATS2 may play central roles in the regulation of anti-tumor immunity.

**Table 4.** Correlations between LATS2 expression and marker genes for tumor-infiltrating immune cells using the TIMER database

		COAD				READ			
Description	Gene markers	None		Purity		None		Purity	
		Cor	*P*	Cor	*P*	Cor	*P*	Cor	*P*
CD8 + T cell	CD8A	0.32	***	0.23	***	0.24	***	0.07	ns
	CD8B PTPRC	0.21 0.54	*** ***	0.16 0.47	** ***	0.18 0.47	* ***	0.05 0.34	ns ***
T cell (general)	CD3D	0.25	***	0.13	**	0.11	ns	−0.06	ns
	CD3E	0.36	***	0.26	***	0.26	***	0.09	ns
	CD2	0.34	***	0.25	***	0.25	**	0.10	ns
B cell	CD19	0.22	***	0.12	*	0.11	ns	0.01	ns
	CD79A	0.31	***	0.20	***	0.22	**	0.04	ns
Monocyte	CD14	0.53	***	0.46	***	0.44	***	0.34	***
	CD115 (CSF1R)	0.57	***	0.50	***	0.54	***	0.44	***
TAM	CD68	0.48	***	0.43	***	0.45	***	0.37	***
	IL10	0.42	***	0.36	***	0.32	***	0.19	*
M1 Macrophage	INOS (NOS2)	−0.14	**	−0.17	***	−0.14	ns	−0.13	ns
	CD80	0.42	***	0.36	***	0.38	***	0.26	**
	IRF5	0.35	***	0.35	***	0.34	***	0.33	***
	IL6	0.39	***	0.33	***	0.40	***	0.36	***
	CD64 (FCGR1A)	0.41	***	0.33	***	0.35	***	0.28	***
M2 Macrophage	CD206	0.48	***	0.41	***	0.46	***	0.33	***
	VSIG4	0.51	***	0.43	***	0.44	***	0.36	***
	MS4A4A	0.52	***	0.45	***	0.52	***	0.45	***
Neutrophils	CD66b	−0.18	***	−0.17	***	−0.21	**	−0.09	ns
	CCR7	0.40	***	0.32	***	0.26	***	0.16	ns
Natural killer cell	KIR2DL1	0.11	*	0.04	ns	0.15	ns	0.10	ns
	KIR2DL3	0.12	*	0.08	ns	0.08	ns	0.01	ns
Dendritic cell Th1 Th2 Tfh Th17 Treg T cell exhaustion	KIR2DL4 KIR3DL1 KIR3DL2 BDCA-1 (CD1C) BDCA-3 (THBD) BDCA-4 (NRP1) IL3RA STAT4 STAT1 T-bet (TBX21) GATA3 STAT6 IL13 IL21 BCL6 STAT3 IL17A FOXP3 CD25 PD-1 (PDCD1) CTLA4	0.18 0.18 0.22 0.37 0.67 0.71 0.52 0.38 0.44 0.36 0.41 0.23 0.29 0.18 0.62 0.46 –0.18 0.46 0.45 0.31 0.39	*** *** *** *** *** *** *** *** *** *** *** *** *** *** *** *** *** *** *** *** ***	0.11 0.11 0.15 0.29 0.64 0.67 0.48 0.29 0.39 0.29 0.36 0.24 0.24 0.13 0.59 0.43 –0.19 0.39 0.36 0.23 0.32	* * ** *** *** *** *** *** *** *** *** *** *** ** *** *** **** *** *** *** ***	0.15 0.05 0.18 0.26 0.70 0.70 0.48 0.30 0.44 0.32 0.45 0.20 0.12 –0.03 0.54 0.42 –0.27 0.49 0.45 0.27 0.39	ns ns * *** *** *** *** *** *** *** *** * ns ns *** *** *** *** *** *** ***	−0.03 –0.04 0.06 0.13 0.64 0.67 0.45 0.17 0.31 0.18 0.38 0.23 0.01 –0.09 0.54 0.39 –0.25 0.39 0.35 0.10 0.23	ns ns ns ns *** *** *** * *** * *** ** ns ns *** *** ** *** *** ns **
	LAG3	0.34	***	0.25	****	0.24	**	0.10	ns

*P < 0.05; **P < 0.01; ***P < 0.001.

### Associations of LATS2 expression and marker genes for immune cells in diverse cancers

**Figure 6.**
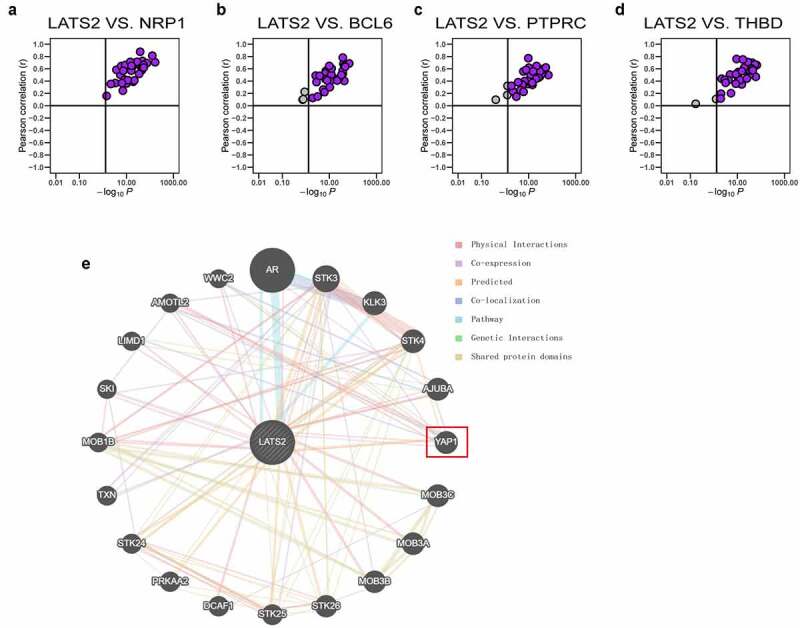
Correlations of LATS2 expression with NRP1, BCL6, PTPRC, and THBD expression in cancer samples and analysis of the PPI network for LATS2. (a–d) LATS2 was positively associated with NRP1, BCL6, PTPRC, and THBD expression in almost all cancers examined, based on data from the TCGA. (e) Protein-protein network view in the GeneMANIA dataset showing the interaction networks of LATS2

We explored whether the associations of LATS2 expression with gene markers for tumor-infiltrating immune cells were also present in other cancers. Specifically, the TCGA datasets were integrated to evaluate the correlations of LATS2 expression with the four specific immune cell gene markers (PTPRC, BCL6, NRP1, and THBD) in 33 cancer types. Strikingly, the results suggested that LATS2 expression and NRP1 expression were positively correlated in all 33 cancers (Figure 6a). LATS2 expression was also positively correlated with BCL6, PTPRC, and THBD expression in the vast majority of cancers (Figure 6(b-d)), indicating that LATS2 may be correlated with some immune infiltrating cells in most cancers. To explore the internal mechanism for LATS2 involvement in cancers, we investigated the PPI network for LATS2 using the GeneMANIA database and found that LATS2 interacted with YAP1 (Figure 6(e)).

## Discussion

The present study revealed that LATS2 mRNA expression and protein expression were decreased in CRC tissues and tumor cell lines by qRT-PCR and TMA immunohistochemistry. We also clarified the relationships between LATS2 expression and the clinicopathologic parameters of CRC patients. Low protein expression of LATS2 was clearly related to poor tumor differentiation, advanced TNM stage, and high T, N, and M stages in CRC. Univariate and multivariate analyses further confirmed that low LATS2 expression and advanced tumor stage could act as independent poor prognostic factors for CRC. In general, CRC patients with high LATS2 expression had longer OS. Therefore, LATS2 may serve as a novel and potential biomarker for prognostic prediction in CRC patients.

Immunotherapy has achieved remarkable progress in recent years and is attracting much attention as a potentially powerful anti-cancer tool. However, the complex mechanisms that regulate tumor immune responses have not been fully clarified. We performed gene enrichment analysis of LATS2 in the present study and found that some inflammation and immune response pathways were activated, including chemokine signaling pathways, JAK-STAT signaling pathways, cytokine-cytokine receptor interaction signaling pathways, and interferon-gamma response signaling pathways. Chemokines participate in homing, circulation, retention, and activation of immune cells and some chemokines are known to cause changes in the tumor microenvironment that allow infiltration of lymphocytes, leading to tumor clearance [22]. Studies have shown that chemokine-chemokine receptor pathways are one of the main signaling pathways for cytotoxic T lymphocyte migration to tumor tissues and initiation of anti-tumor activity [23,24]. In addition, interferon-gamma can recruit macrophages and cytotoxic CD8 + T cells or directly inhibit the proliferation of Th2 cells, thereby exerting effective anti-tumor effects [25]. The present findings suggest that LATS2 may promote the immune response in CRC through various pathways.

The development of an effective anti-tumor immune response depends on the synergistic effects of immune cells. Thus, the relationships between LATS2 expression and tumor-infiltrating immune cells were assessed by TIMER and ssGSEA. Notably, LATS2 expression had positive correlations with tumor-infiltrating immune cells, indicating that LATS2 may be associated with tumor immune infiltration in CRC. Tumor-infiltrating immune cells have a pivotal role in the inhibition or promotion of tumor progression. Accumulating clinical and experimental data demonstrated that tumor-infiltrating lymphocytes in tongue squamous cell carcinoma [26], lung cancer [27], and CRC [28] inhibited tumor growth and prolonged survival time. In the present study, we found that four specific gene markers for immune cells (PTPRC, BCL6, NRP1, and THBD) were significantly positively correlated with LATS2 expression. These findings are similar to the notion that downregulation of LATS2 promotes apoptosis of T cells in ovarian cancer [13]. Recent studies revealed that T-cell-infiltrated neuroblastomas were enriched with dendritic cells, and that the abundance of these cells was related to favorable clinical outcomes in neuroblastoma [29]. Meanwhile, activated CD8 + T cells and high densities of tumor-infiltrating CD4+ Th1 cells indicated prolonged survival in non-small cell lung cancer [30]. Furthermore, LATS2 expression was positively correlated with the expression of PTPRC, BCL6, NRP1, and THBD in almost all cancers in the TCGA database. Taken together, these findings suggest that LATS2 is specifically correlated with immune infiltrating cells in CRC.

Moreover, when combining the PPI network for LATS2 with recent study findings, YAP1 caught our attention. YAP1, a core molecule in the Hippo pathway, can be phosphorylated by LATS2 to limit its nuclear translocation [31]. A recent study revealed the novel mechanism that downregulation of LATS2 resulted in increased YAP1 translocation and up-regulated PD-L1 expression, finally leading to immune suppression in ovarian cancer [13]. In addition, activation of YAP exhausted CD8 + T cell-mediated immunity and upregulated PD-L1 in malignant pleural mesothelioma [32]. Therefore, interactions between LATS2 and YAP1 may be a potential mechanism for regulation of the tumor microenvironment in CRC, although further studies are necessary to confirm this hypothesis.

There are several limitations in our research. First, the size and quality of our clinical samples were limited, and larger-scale studies are needed to verify our findings. Second, the underlying mechanisms for the association of LATS2 with tumor-infiltrating immune cells in CRC were not revealed. Further studies are warranted to determine the underlying mechanisms for the correlations of LATS2 with tumor-infiltrating immune cells in CRC.

## Conclusion

In conclusion, we have shown that LATS2 was decreased in CRC. Increased LATS2 expression was correlated with favorable clinical outcomes and LATS2 may represent an independent clinicopathological marker to evaluate the development, metastasis, and prognosis of CRC. Furthermore, LATS2 was positively correlated with tumor-infiltrating immune cells. Therefore, we speculate that LATS2 is a possible prognostic and immunological biomarker for CRC. These findings provide new insights for investigating the potential functions and effects of LATS2 in tumorigenesis and tumor immunology.

## Supplementary Material

Supplemental Material

## Data Availability

The gene expression data of CRC and normal tissues were extracted from the TCGA Data Portal (https://tcga-data.nci.nih.gov/tcga). All the data will be provided on reasonable request from the corresponding author.

## Highlights

- LATS2 is down-regulated in CRC and related to tumor differentiation and TNM stage.
- Decreased LATS2 expression is correlated with unfavorable clinical outcomes.
- LATS2 is involved in immune-related pathway regulation and positively related to tumor-infiltrating immune cells.
