# Supplementary material for: Large tumor suppressor 2 is a prognostic biomarker and correlated with immune infiltrates in colorectal cancer
Source: Bioengineered. 2021 Dec 19;12(2):11648–61. doi: 10.1080/21655979.2021.1996513 (PMC8810027; doi:10.1080/21655979.2021.1996513)
Supplement: Supplemental Material [file KBIE_A_1996513_SM5632.zip › Revised Table S1.docx]

**Table S1.** Primers for qRT-PCR in this study.

| **Gene** | **Primers (5′-3′)** |
| --- | --- |
| LATS2-F | GGGCTTCATCCACCGAGACA |
| LATS2-R | GCCCTCTGCTCTAGGGTCTT |
| GAPDH-F | ACAACTTTGGTATCGTGGAAGG |
| GAPDH-R | GCCATCACGCCACAGTTTC |
