# Supplementary figures and images for: Large tumor suppressor 2 is a prognostic biomarker and correlated with immune infiltrates in colorectal cancer
Source: Bioengineered. 2021 Dec 19;12(2):11648–61. doi: 10.1080/21655979.2021.1996513 (PMC8810027; doi:10.1080/21655979.2021.1996513)

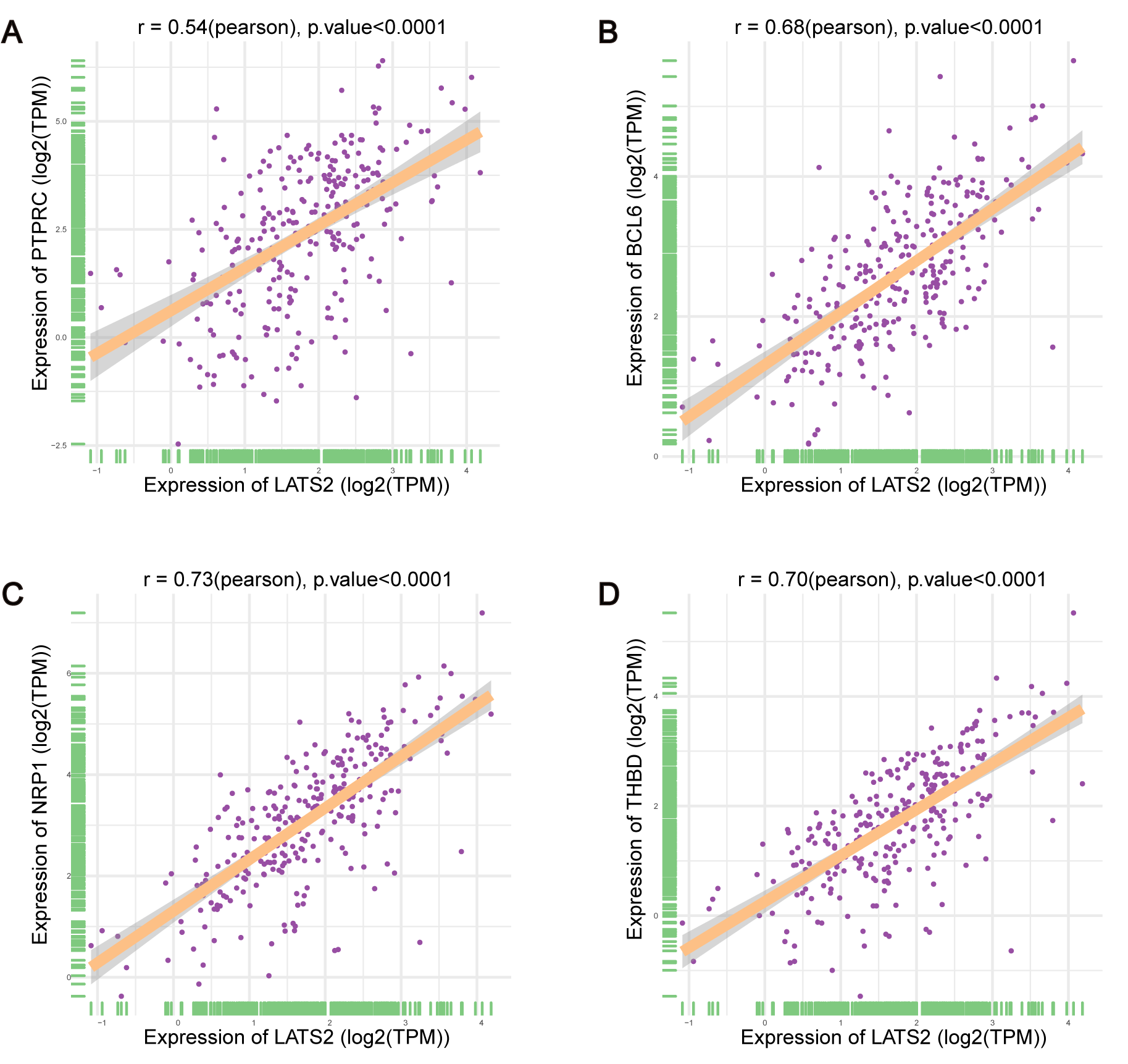

Supplement: Supplemental Material [file KBIE_A_1996513_SM5632.zip › Supplementary Figure 1.tif]
